# Biotechnological Research Progress in Jatropha, a Biodiesel-Yielding Plant

**DOI:** 10.3390/plants11101292

**Published:** 2022-05-12

**Authors:** Jameel M. Al-Khayri, Wudali N. Sudheer, Thenmozhi R. Preetha, Praveen Nagella, Adel A. Rezk, Wael F. Shehata

**Affiliations:** 1 Department of Agricultural Biotechnology, College of Agriculture and Food Sciences, King Faisal University, Al-Ahsa 31982, Saudi Arabia; arazk@kfu.edu.sa (A.A.R.); wshehata@kfu.edu.sa (W.F.S.); 2 Department of Life Sciences, CHRIST (Deemed to be University), Bengaluru 560029, India; wudali.sudheer@res.christuniversity.in (W.N.S.); rt.preetha@science.christuniversity.in (T.R.P.); 3 Agricultural Research Center, Virus and Phytoplasma Research Department, Plant Pathology Research Institute, Giza 12619, Egypt

**Keywords:** *Jatropha curcas*, biodiesel, natural resource, micropropagation, plant genetic transformation

## Abstract

Environmental pollution is one of the most pressing challenges in today’s world. The main cause of this pollution is fuel emissions from automobiles and other sources. As industrialization progresses, we will be unable to compromise on the use of energy to power heavy machines and will be forced to seek out the best options. As a consequence, utilizing green fuel, such as biodiesel derived from natural sources, is a realistic option. *Jatropha curcas* L. (Euphorbiaceae) is recognized as the greatest feedstock for biodiesel production throughout the world, and it has gained a huge market value in the recent years. Conventional cultivation alone will not be sufficient to meet the global need for the plant’s biomass for the production of biodiesel. Adoption of plant tissue culture techniques that improve the biomass availability is an immediate need. The present review provides detailed information regarding in-vitro plant propagation (direct and indirect organogenesis), somatic embryogenesis, and acclimatization protocols of plantlets for stabilized production of biomass. The review also focuses on biotechnological approaches such as gene transformation studies, production of haploids, and double haploids for developing elite germplasm for high biomass and improved traits for the production of biodiesel.

## 1. Introduction

*Jatropha curcas* L. is a non-edible, oil-bearing, and zero waste perennial shrub or small tree belonging to the Euphorbiaceae family. The plant is commonly known by several names, such as Barbados nut, termite plant, fig nut, black vomit nut, curcas bean, physic nut, and purge nut. The plant grows well in tropical and sub-tropical climates with a variety of medicinal properties in its oil [1]. Its rapid growth, hardness, and easy propagation make it grow under a wide range of rainfall regimes and harsh climatic conditions; thus, this species has spread far beyond its original distribution. The height of the plant can range from three to five meters, and under favorable conditions it can grow up to 10 m. The plant exhibits articulated growth, a straight trunk, and thick greenish-bronze-colored branches with a soft wood [2]. The plant has a well-established taproot system with four shallow lateral roots. This type of root system aids in the prevention of soil erosion [3]. The leaves are 10–15 cm long, five-lobed and heart-shaped, with simple, smooth margins that are cordate at the base and acute at the apex [4,5]. Jatropha is a monoecious plant with male and female flower ratio of approximately 29:1 [6]. The flowers are yellowish-green, arranged in axillary clusters. The inflorescence is complex, with the main and co-fluorescence formed terminally on branches. The fruits are 3–4 cm long, ellipsoidal, and trilocular. Each fruit contains three black, oblong, large seeds [1,5] weighing approximately 0.53 to 0.86 g. The seed kernel is composed of 57–63% of lipids and 22–27% of protein [1]. Approximately 4–40% of the viscous curcas oil can be extracted from its seeds [7]. The life span of the plant is found to be about 50 years [2].

Oil has played an important role in recent advancements and economic development, as it is the most useful source of essential energy. It plays a key role in the progress of industry, agriculture, and transportation. With the rise in population, the need for petroleum products has increased, and thus the production rate has elevated, leading to the depletion of the world petroleum reserves and heightened environmental concerns [8]. The search for alternate sources for petroleum-based fuel has been triggered due to these reasons. Usage of biodiesel as an alternative form of energy has increased recently. According to Energy Information Administration (EIA) reports, the US consumed 43 million barrels of biodiesel, which is expected to increase in the coming decades (https://www.eia.gov/, accessed on 13 March 2022). Biodiesel based on biomass is one of the most appealing strategies, in which fatty acid methyl esters from vegetable oil can be considered to be the best substitute for diesel [9]. Biodiesel extracted from Jatropha, a non-edible vegetable oil source, is a cheap and also the best renewable alternative to conventional diesel. By 2030, it is expected that Mexico alone will be equipped to produce 255.75 metric tons of jatropha biomass for biodiesel production (https://www.statista.com/, accessed on 13 March 2022).

The oil obtained from *Jatropha curcas* was used as lamp oil and in the production of soap for centuries in Portugal [10]. The oil contains approximately 97.6% neutral lipids, 0.95% glycolipids, and 1.45% phospholipids. The unsaturated fatty acids are predominantly higher than saturated fatty acids. This oil can also be used as a substitute for fossil fuel, as it has 41.5 to 48.8% of oleic acid, 34.6 to 44.4% of linoleic acid, 10.5 to 13% of palmitic acid, 2.3 to 2.8% of stearic acid, along with cis-11-eicosadienoic and cis-11,14-eicosadienoic acids as the main fatty acids. This oil, when used as fossil fuel, could also help to decrease the emissions of greenhouse gases [11]. Apart from its use as fossil fuel, this oil is also used in the manufacture of candles, soaps, and cosmetics. After the extraction of oil from the seeds, the seed cake can be detoxified and can be used as animal feed, as it is highly nutritious and can supplement protein [12]. The by-products after the oil extraction can also be used for the production of cellulosic methanol [13].

Along with their economic value, jatropha species are also regarded as rich sources of phytochemicals such as terpenes, cyclic peptides, alkaloids, and lignans. Diterpenes (phorbol esters, dinorditerpenes, deoxypreussomerins, and pimarane, lathyrane and rhamnofolane); sesquiterpenoids and triterpenes (taraxasterol, β-amyrin, and β-sitosterol, (Z)-3-O-coumaroyloleanolic, stigmasterol and daucasterol, friedelin); alkaloids (pyrrolidine (5- hydroxypyrrolidin-2-one) and pyrimidine-2,4-dione (uracil), imidazole, diamide (curcamide)); flavonoids (flavonoid glycoside I and flavonoid glycoside II, nobiletin, tomentin); phenolics (3-hydroxy-4-methoxybenzaldehyde and 3-methoxy-4-hydroxybenzoate acid, caffeoylaldehyde and syringaldehyde); lignans, neolignans, coumarins, coumarino-lignoids, and phytosterols are some of the most studied phytochemicals of the plant [2]. There are many medicinal and pharmacological effects exhibited by the plant due to the presence of these varieties of phytochemicals. Some of the popular and well-studied pharmacological activities include anti-inflammatory effects, antimicrobial properties, anti-oxidant, anti-cancer, antiviral, anti-diabetic, analgesic activity, hepatoprotective activity, wound healing activity, anticoagulant, and procoagulant activity [2].

Jatropha can be sexually propagated via seeds and vegetatively propagated through stem cuttings. Stem cuttings are usually preferred over seeds for propagation. In nurseries, cuttings are prepared from one-year-old terminal branches inoculated with mycorrhizal fungi to improve symbiosis in field conditions. Under tropical, humid conditions, fruiting lasts for four months per year and can be harvested thrice. Some of the major factors that affect seed production and oil yield are reduced branching, low female flower count, inadequate pollination, and poor soil quality. [1]. Vegetative propagation of jatropha is reported to show a low seed set. It was also observed that vegetative propagation cannot form deep-rooted plants that can be easily uprooted [7]. Thus, numerous studies on tissue culture of jatropha, along with genetic manipulation, have been extensively done due to their many beneficial applications. The present review focuses on the tissue culture aspects for in-vitro propagation of *J. curcas* through direct and indirect organogenesis and somatic embryogenesis. The review also presents advanced biotechnological approaches, such as production of haploids, double haploids, and genetic transformation studies for the production of elite germplasms that aid in establishing sustainability for biomass production.

## 2. Regeneration Studies

For a sustained supply of biomass for biodiesel generation, in-vitro growth and multiplication of jatropha is important. Tissue culture methods were developed utilizing a variety of methodologies that included the use of different plant growth regulators and explants, and they showed the potential for large-scale production to ramp up the production of biodiesel.

### 2.1. Direct Organogenesis

**Figure 1:**
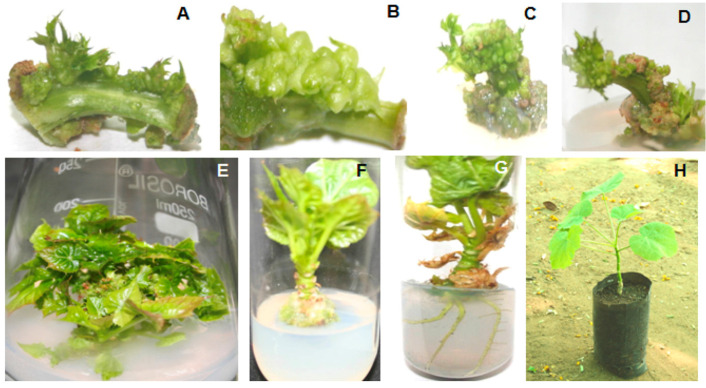
Direct organogenesis of jatropha from petiole explants. (**A**) in vitro petiole in horizontal position, (**B**) in vivo petiole in horizontal position, (**C**) in vitro petiole in vertical position and (**D**) in vivo petiole in vertical position on MS medium supplemented with 2.27 µM TDZ. (**E**) Shoot proliferation of induced shoot buds on MS medium supplemented with 10 µM KN + 4.5 µM BAP + 5.5 µM NAA. (**F**) Elongation of proliferated shoot on MS medium supplemented with 2.25 µM BAP and 8.5 µM IAA. (**G**) Development of roots at the base of elongated shoot on half strength of MS medium supplemented with 5 µM IBA + 5.7 µM IAA + 11.0 µM NAA after 4 weeks. (**H**) Regenerated plant in polybag. (Source: Kumar and Reddy 2012: https://doi.org/10.1016/j.indcrop.2012.02.011, accessed on 2 February 2022; reproduced with permission from the publisher; License No: 5267680531691).

Direct organogenesis is characterized by the induction and proliferation of organs directly from the surface of explants. Explants from various parts of the jatropha were used for direct organogenesis, and the studies reveal that the response was favorable. Murashige and Skoog (MS) media supplemented with various types of auxins and cytokinins induced shooting when leaf explants were inoculated. The first attempt of regeneration studies in jatropha was carried out by Sujatha and Mukta (1996), in which leaf explants served as a better source when cultured on 6-Benzylaminopurine (BAP) along with Indole-3-butyric acid (IBA) helped in inducing shoots [14]. Sujatha et al., (2005) reported that in-vitro shoots sub-cultured on MS medium supplemented with 8.9 µM BAP and 2.5 µM IBA gave better results in terms of proliferation and multiplication [15]. Thidiazuron (TDZ), a cytokinin, has shown effective regeneration abilities with varied explant sources for direct organogenesis of jatropha. Kumar and Reddy (2012) reported that TDZ supplemented medium was optimal for regeneration of jatropha from petiole explants (Figure 1), in which MS medium supplemented with 2.27 µM TDZ exhibited a 51.19 percent response [16]. Similarly, leaf explants, when exposed to 20 mg/L TDZ for 20 min, induced 65 to 87 percentage response for shooting when cultured on basal MS media [17,18]. N-(2-chloro-4-pyridyl)-N-phenyl urea (CPPU) was employed for the induction of shoots in addition to the most regularly used plant growth regulators. Singh (2017) reported that MS medium supplemented with 0.5 mg/L CPPU induced shoots with 68.1% response [19]. Scientific studies have suggested that cytokinins like BAP and TDZ, alone or in combination with other plant growth regulators, favors shoot induction and also aids in shoot proliferation. Similarly, MS media fortified with varying concentrations of auxins like IBA individually or in combination with other auxins, such as Indole-3-acetic acid (IAA) and 1-Naphthaleneacetic acid (NAA), have been reported to be ideal for rooting (Table 1).

When cotyledon explants were inoculated onto MS medium supplemented with BAP, they showed a significant response for induction of shoots [20]. MS medium supplemented with 1.0 mg/L BAP, 0.1 mg/L IBA, and 0.5 mg/L TDZ showed 78.42 percentage shooting. Similarly, cotyledonary explants, when cultured on ½ strength MS medium supplemented with 4.4 µM BAP and 2.8 µM IAA, regenerated shoots [21]. Liu et al., (2016) induced shoots with a higher percentage of shoot response (88.42%) from cotyledon explants by culturing them onto basal MS medium with an exposure of explant to 20 mg/L TDZ [22], and Kumar et al., (2011b) reported that MS medium supplemented with 9.08 µM TDZ induced shoots [23]. Similarly, when hypocotyl explants were inoculated onto MS media supplemented with 1.0 mg/L TDZ, they showed a maximum response (92.9%) for shooting [24]. Different concentrations and combinations of auxins and cytokinins used for regeneration of *J. curcas* L. and their responses are presented in Table 1.

### 2.2. Micropropagation/Multiplication from Preformed Meristems

Shoot tips and meristems were also employed for induction of shoots in addition to leaf and cotyledons. Explants of shoot tips, when cultured on MS media supplemented with 1.0 mg/L BAP and 0.5 mg/L IAA, induced 3.45 shoots per explants [25]. Imtiaz et al., (2014) induced shoots from shoot tips as explant on MS medium augmented with 8.88 µM BAP/13.32 µM BAP + 4.92 µM IBA [26]. MS medium supplemented with 0.5 mg/L 2-isopentenyl adenine (2-iP) showed 90% response for induction of shoots from meristems as explants [27]. Generally, nodal explants are being considered as an optimal source for regeneration as there is presence of axillary buds in the axils of nodes. In the majority of instances, they have been considered as efficient sources for regeneration. Nodal segments, in addition to shoot tips and meristems, showed the potential for direct organogenesis. According to Shrivastava and Banerjee (2008), MS media supplemented with 3.0 mg/L BAP and 1.0 mg/L IBA resulted in the induction of 100 shoots from a single explant [28]. Nodal explants, when cultured onto MS medium supplemented with 1.0 mg/L BAP and 0.5 mg/L IBA, showed a higher response of 86–90% for shooting [29]. Shoots were induced from axillary buds when cultured onto MS media supplemented with 2.8 µM IAA and 13.93 µM Kinetin (KN) [30]. Similarly, MS media supplemented with 2.0 mg/L BAP, 1.0 mg/L KN, and 0.1 mg/L Gibberellic acid (GA_3_) also induced shoots [31].

**Table 1:** Direct organogenesis from various explants of *Jatropha curcas* L.

Explant	Shooting Media	Response Percentage	No. of Shoots	Rooting Media	Percentage of Acclimatization	Reference
Leaf	MS + 2.22 µM BAP + 4.9 µM IBA	45	50	Full Strength MS medium	More than 80%	[14]
Leaf	MS + 8.88 µM BAP	-	8.3	-	-	[32]
Leaf	MS + 22.2 µM BAP + 4.9 µM IBA Subculture medium: 8.9 µM BAP + 2.5 µM IBA	90	-	MS + 5.4 µM NAA	-	[15]
Leaf	Shoot induction medium: Liq. MS + 2.0 mg/L KN. Proliferation medium: Liq. MS + 1.5 mg/L BAP + 0.5 mg/L IAA + 0.2 mg/L KN	92.1 ± 3.1%	-	Ex-vitro rooting (76.4% rooting efficiency)	More than 90%	[33]
Leaf	Induction medium: MS + 1.0 mg/L TDZ + 0.5 mg/L KN + 0.5 mg/L GA_3_. Proliferation medium: MS + 0.3 mg/L BAP + 0.01 mg/L IBA.	Shoot bud induction rate: 81.48 ± 3.21%. Shoot elongation rate: 75.72 ± 3.85%	3.51 ± 0.78	½ MS + 2.0 mg/L IBA	-	[34]
Leaf	Shoot induction medium: MS + 0.5 mg/L CPPU. Shoot proliferation medium: 0.5 mg/L BAP + 1.0 mg/L IAA + 0.5 GA_3_	68.1%	More than 25 buds per explant	Ex-vitro rooting	-	[19]
Petiole	Shoot induction medium: MS + 2.27 µM TDZ. Proliferation medium: MS + 10 µM KN + 4.5 µM BAP + 5.5 µM NAA. Shoot elongation medium: MS + 2.25 µM BAP + 8.5 µM IAA.	51.19%	9.75 buds per explant	½ MS + 5 µM IBA + 5.7 µM IAA + 11 µM NAA	More than 90%	[16]
Petiole	Initiation medium: MS + 2.27 µM TDZ. Proliferation medium: MS+ 10 µM KN + 4.5 µM BAP + 5.5 µM NAA. Shoot elongation medium: MS + 2.25 µM BAP + 8.5 µM IAA.	57.61%	4.98 shoot buds per explant	½ MS + 15 µM IBA + 11.4 µM IAA + 5.5 µM NAA.	More than 90%	[35]
Petiole	Initiation medium: 20.0 mg/L TDZ solution for 20 min, later inoculated on Basal MS medium. Elongation medium: MS + 0.4 mg/L GA_3_.	65.78%	6.77 shoot buds per explant	0.3 mg/L IBA + 16.0 mg/L L-glutamine (gln).	-	[17]
Petiole	Initiation medium: 20.0 mg/L TDZ solution for 20 min, later inoculated on Basal MS medium. Multiplication medium: Shoots were grafted on seedling stocks and cultured on MS + 0.1 mg/L IBA + 2.0 mg/L sodium nitroprusside.	87.35%	10.48 ± 0.42 buds per explant	½ MS + 2.0 mg/L sodium nitroprusside	-	[18]
Cotyledons	½ MS + 4.4 µM BAP + 2.8 µM IAA	-	4.8	-	-	[21]
Cotyledonary petiole	Initiation medium: 20.0 mg/L TDZ solution for 20 min, later inoculated on basal MS medium. Elongation medium: MS + 7.5 mg/L arginine.	88.42%	12.67	½ MS + 0.1 mg/L IBA.	-	[22]
In-vitro cotyledonary leaf	Initiation medium: MS + 9.08 µM TDZ. Proliferation medium: 10.0 µM KN + 4.5 µM BAP + 5.5 µM NAA. Shoot elongation medium: MS + 2.25 µM BAP + 8.5 µM IAA.	81.07 ± 8.26%	20.17	½ MS + 1 5.0 µM IBA + 5.7 µM IAA + 5.5 µM NAA	More than 90%	[23]
Cotyledons	MS + 1.0 mg/L BAP + 0.1 mg/L IBA + 0.5 mg/L TDZ	78.42 ± 10.28%	-	½ MS + 0.2 mg/L IBA	-	[20]
Hypocotyl	Initiation medium: MS + 1.0 mg/L TDZ. Elongation medium: MS + 2.0 mg/L KN + 1.0 mg/L BAP. Proliferation medium: 1.5 mg/L IAA + 0.5 mg/L BAP.	92.9 ± 2.10%	-	½ MS + 3.0 mg/L IBA + 1.0 mg/L IAA + 1.0 mg/L NAA	~90%	[24]
Shoot tip	MS + 0.5 mg/L IAA.	90%	3.44 ± 0.17	½ MS + 3.0 mg/L IBA	60–70%	[36]
Shoot tip	MS + 1.0 mg/L BAP + 0.5 mg/L IAA	-	3.45 ± 0.73	-	-	[25]
Shoot tip	Induction medium: MS + 13.32 µM BAP + 4.92 µM IBA. Multiplication medium: MS + 13.32 µM BAP + 4.92 µM IBA.	93.33%	6.7	½ MS + 14.76 µM IBA (4.93 roots per culture)	-	[26]
Apical shoot	MS + 4.44 µM BAP	-	3.9 ± 0.27	½ MS + 4.90 µM IBA (85.71% rooting)	82%	[37]
Meristem	MS + 0.5 mg/L 2-iP	90 ± 0.06%	-	-	-	[27]
Node	MS + 4.5 µM TDZ + 8.9 µM IBA	-	12.3 ± 1.7	½ MS + 5.4 µM NAA	-	[15]
Node	MS + 3.0 mg/L BAP + 1.0 mg/L IBA + 25 mg/L adenine sulphate + 50.0 mg/L glutamine + 15.0 mg/L L-arginine + 25.0 mg/L citric acid.	-	10.0 + 1.30	½ MS + 3.0 mg/L IBA	100%	[28]
Node	MS + 0.5 mg/L BAP + 0.1 mg/L IBA + 10.0 mg/L adenine sulphate + 15.0 mg/L L-glutamine and L-arginine + 50.0 mg/L Augmentin + 15.0 mg/L coconut water.	-	5	½ strength of same shooting medium + 0.5 mg/L IBA (85% response for rooting)	100%	[38]
Node	MS + 3.0 mg/L BAP + 1.0 mg/L IBA.	-	5 to 6	½ MS + 3.0 mg/L IBA	95%	[39]
Node	MS + 8.0 µM BAP + 2.0 µM IBA + 45 µM Adenine sulphate (AdS) + 15.0 µM glutamine + 10 µM proline.	-	9.8 ± 0.84	½ MS + 2.0 µM IBA	80%	[40]
Node	Shoot induction medium: 0.5 mg/L BAP + 0.5 mg/L IBA.	86–90%	6.00 ± 0.31	MS + 0.25 mg/L IBA	66.67–86.67%	[29]
Nodal segments	Induction medium: 2.0 mg/L BAP + 1 mg/L IAA. Multiplication medium: 0.5 mg/L BAP + 0.5 mg/L IAA.	96.67 ± 3.33%	1.73 ± 0.07 shoot buds on initiation medium, 9.33 ± 0.09 shoot buds on multiplication medium	½ MS + 3.0 mg/L IBA (73.33 ± 3.33% rooting)	More than 80%	[41]
Axillary node	MS + 2.8 µM IAA + 13.93 µM KN.	83 ± 0.6%	-	MS + 14.7 µM IBA	-	[30]
Axillary bud derived shoots	MS + 2.22 µM BAP + 0.049 µM IBA	-	5.9 ± 0.93	MS + 4.90 µM IBA	82%	[37]
Axillary bud	Shoot induction medium: MS + 2.0 mg/L BAP + 1.0 mg/L KN + 0.10 mg/L GA_3_. Multiplication medium: MS + 2.0 mg/L BAP + 1.0 mg/L KN + 0.05 mg/L GA_3_.	91.65 ± 0.31%	10.24 ± 0.07	MS + 1.50 mg/L IBA + 0.10.0 mg/L NAA	80%	[31]

### 2.3. Indirect Organogenesis

Indirect organogenesis is characterized by the induction and proliferation of callus followed by shoots and root development from the callus surface. Studies have revealed that leaf as an explant showed more potential for establishment of callus when compared to other explant sources (Table 2). Leaf explants cultured on MS media supplemented with 1.0 mg/L NAA and 5.0 mg/L BAP induced a green compact callus. Multiple shoots (12.62) were induced by subculturing the derived callus on MS media supplemented with 1.5 mg/L BAP and 0.5 mg/L IBA [42]. Verma et al., (2016) induced callus from leaf explants when cultured on MS medium supplemented with 0.5 mg/L of NAA and 0.25 mg/L BAP, and subsequently 70% response of shoot proliferation was observed [43]. Combinations of BAP and IBA at various concentrations has induced callus followed by shoot induction when explant sources like leaf, node, and embryo were cultured. Boonyanan (2021) reported that MS media supplemented with 2.0 mg/L BAP along with 1.0 mg/L IBA induced callus from leaf explant and further subculturing of callus onto BAP enriched medium resulted in the formation of multiple shoots with 43.7% response [44]. Embryos from jatropha were inoculated on MS medium supplemented with 1.5 mg/L BAP and 1.0 mg/L IBA induced callus; further subculturing on the same medium induced multiple shoots [39]. Similarly, nodal explants were cultured on media supplemented with 8.0 µM BAP and 2.0 µM IBA induced callus, and subsequently multiple shoots were obtained [40]. Hegazi et al., (2020) reported that 0.45 µM TDZ supplemented medium also induced callus and multiple shoots when cotyledons were used as explants [45]. MS medium supplemented with varied concentrations of IBA have been found to be potential auxin for rooting of the in-vitro raised plantlets.

### 2.4. Somatic Embryogenesis

Somatic embryogenesis is one of the biotechnological advancements in which an embryo is developed from the somatic tissues/explants by proving the totipotent nature of the cells [50]. This technique helps us to derive the plants with desired characteristics, be it disease resistance or high yields [51]. In jatropha, somatic embryogenesis was established using various explants through the tissue culture technique.

Studies revealed that various concentrations and combinations of auxins and cytokinins when supplemented in growth medium resulted in the formation of various types of embryos from somatic explants (Table 3). Green embryogenic callus was induced from leaf and shoot tip explants when inoculated onto MS medium supplemented with 0.5 mg/L 2,4-D and 5.0 mg/L BAP. It was also observed that plant conversion rate is approximately 54% for shoot tips and 51% for leaf explants [52]. Jha et al., (2007) reported that 80% frequency for response in terms of development of globular somatic embryos was recorded from leaf explants when inoculated onto MS medium supplemented with 2.3 µM KN along with 1.0 µM IBA and 13.6 µM AdS [53]. Similarly, Cai et al., (2011) reported that 0.1 to 0.2 mg/L 2,4-D fortified MS medium showed highest frequency of somatic embryos development from immature zygotic embryos as explant [54]. Along with MS medium, Y3 medium (Y3 minerals medium) supplemented with 0.5 mg/L 2,4-D along with 0.5 mg/L BAP induced somatic embryos from petiole explants of jatropha, with a 100 percent success rate [55]. Globular embryos were developed from cotyledon explants when cultured on MS medium supplemented with 2.0 mg/L BAP [56] and also 1.0 mg/L picloram supplemented medium, resulting in the formation of somatic embryos from the same cotyledonary explants [57].

## 3. Acclimatization of Plantlets

The acclimatization procedure creates a stress-free environment for in-vitro grown plantlets. This mechanism aids the plantlets in overcoming the harshness of the environment and establishing themselves successfully. Microbial exposure, humidity differences, light, and temperature are the critical factors that influence plantlet survival. Therefore, these factors have to be taken into consideration before transferring the plantlets from in-vitro conditions to the field environment [60]. Continuous transpiration in the field causes stress in plantlets. Hence, including anti-transpirants into the acclimatization media aids in plantlet survival [61].

There are many studies that suggested various acclimatization media for better survival rate of in-vitro grown jatropha plantlets. Soil rite, cocopeat, compost, garden soil, vermiculite, sand, and manure are some of the components used in the acclimatization medium for successful establishment of plantlets. Table 4 presents various media used for acclimatization of regenerated jatropha plantlets.

## 4. Genetic Transformation Studies of *Jatropha curcas* L.

Plant genetic transformation is a widely used tool for the generation of transgenic plants with a required specific trait. Gene transformation permits the introduction of a useful gene from one organism into another, with the subsequent stable integration and expression of the introduced foreign gene. This plays a significant role in plant breeding programs by producing novel genetically diverse plant materials. The transformation or gene delivery methods include: electroporation, lipofection, microinjection, sonication, particle bombardment, silicon carbide mediate transformation, laser beam mediated transformation, agrobacterium-mediated method, and virus-based methods [64]. *Agrobacterium* mediated transformation is widely used and is preferred over other methods due to its simplicity, cost-effectiveness, lesser rearrangements of the transgene, and most importantly, its ability to transfer relatively larger DNA segments and integration of foreign genes into transcriptionally active regions [65].

Li et al., (2008) was the first to perform agrobacterium-mediated transformation using cotyledonary disc as explant. The transformation was performed using the LBA4404 strain, and phosphinothricin was used for selection. They observed that approximately 55% of the cotyledonary explants produced phosphinothricin-resistant callus on the MS medium. The transformants were detected by β-glucuronidase activity and confirmed using PCR and southern hybridization analysis. Of the total inoculated explants, 13% were found to produce transgenic plants after four months [66]. Kumar et al., (2010) studied various factors that would influence agrobacterium-mediated transformation of *J. curcas* using leaf explants. The LBA4404 strain of agrobacterium harbouring binary vector pCAMBIA 1304 with sense-dehydration responsive element binding (S-DREB2A), β-glucuronidase (gus), and hygromycin-phosphotransferase (hpt) genes were transformed into jatropha. The highest stable transformation efficiency of 29% was achieved when four- day precultured, non-wounded explants were infected with the agrobacterium culture and co-cultivation with acetosyringone on MS medium. The transformation was confirmed using GUS histochemical analysis. The presence of the transgene was confirmed using PCR and DNA gel blot hybridization [67].

As discussed earlier, development of elite germplasm is a major goal of gene transformation studies. Salinity can impact the growth and yield of jatropha. Jha et al., (2013) developed transgenic jatropha plants with the SbNHX1 gene (*Salicornia brachiate* vacuolar Na+/H+ antiporter gene) using microprojectile bombardment mediated transformation. They confirmed the transgene integration by PCR and RT-PCR methods. Real-time qPCR was used to determine the copy number. The developed transgenic lines were reported to show salt tolerance up to 200 mM NaCl, which was better in comparison with the wild type [68]. The plant breeding program has significantly enhanced agricultural productivity through the development of high-yielding crop varieties. Many traits could be targeted for the improvement of *J. curcas*, such as increasing the flower and fruit production, seed quality (size, oil content, and oil component), etc. [69,70]. The genome size of *J. curcas* is relatively small and is organized within 22 chromosomes (2n) [71]. Ha et al. reported that the whole genome size of *J. curcas* using PacBio and Illumina platforms was approximately 339 Mbps [72]. The smaller genome size of *J. curcas* has many advantages, including easy genetic transformation and short generation duration. Jatropha has become one of the most attractive model plants for wood energy and genome analysis among the family Euphorbiaceae [71].

The effect of endogenous cytokinins treatment on the flower development in *J. curcas* was studied by Ming et al., (2020) through transgenic expression of cytokinin biosynthetic gene AtIPT4 under the control of JcTM6 (*J. curcas* tomato mads box gene 6) promoter that is mostly active in flowers. They found an increase in the number of flowers in a single inflorescence, but both the male and the bisexual flowers were infertile due to the continuous expression of the transgene. The transformation was performed using *A. tumefaciens* EHA105 [73].

Transgenic jatropha producing enlarged seeds were successfully developed by transformation methods. Chacuttayapong et al., (2021) transformed jatropha using genes for the larger seed size found via the rice FOX-hunting system, identified as the genes LOC_Os03g49180 (Os03), LOC_Os04g43210 (Os04), LOC_Os08g41910 (Os08), and LOC_Os10g40934 (Os10). Rice FOX-hunting system was established by the introduction of rice full-length cDNA into Arabidopsis plants by *A. tumefaciens* mediated transformation. The LOC_Os03g49180 gene encodes for ceramidase enzyme that hydrolyses ceramide into sphingosine and fatty acids. Sphingolipids are reported to be important in the kernel development of sunflower seeds. In the study conducted by Chacuttayapong et al., (2021), two types of overexpressing constructs were developed using LOC_Os10g40934.3 and LOC_Os10g40934.11. Transgenic jatropha was produced from excised shoots by using auxins for promoting root formation (kept under dark) and delaying the timing of antibiotic selection in cultivation media [70].

The main component of the jatropha seed storage oil is triacylglycerol (TAG). TAGs contain C16 or C18 fatty acid chains that are covalently linked to glycerol. This makes TAGs a high energy source for seed germination, seedling growth, and development. TAGs are synthesized through the Kennedy pathway. Diacylglycerol acyltransferase (DGAT) and phospholipid: diacylglycerol acyltransferase (PDAT) are the key enzymes involved in TAG biosynthesis in Arabidopsis. TAG biosynthesis could be upregulated by overexpression of the DGAT1 gene [72,74]. Maravi et al., (2016) developed transgenic jatropha by ectopically expressing *Arabidopsis DGAT1* gene (*AtDGAT1*) via *Agrobacterium*-mediated transformation, and it was found to have increased oil content (TAG and Diacylglycerols (DAG)) by 20–30% in seeds and 1.5 to 2.0-fold increase in leaves. They also observed an increase in the plant height, seeds per tree, seed length and breadth, and average seed weight of the transgenic plant in comparison with the wild type [74]. Gene expression and homology modelling studies revealed that PDAT homolog Jatcu.04g000545 has higher expression levels at all stages than DGAT homolog Jatcu.04g000511, indicating that TAG biosynthesis in jatropha is mainly catalyzed by PDAT [72[p]. Studies carried out by Arockiasamy et al., (2021) made information available for both phenotype and genotype of jatropha, assisting in identification of quantitative trait locus (QTLs). They also established a genetic transformation approach using cotyledonary leaves, with a transformation rate of 10–12% and molecular characterization of 70 transgenic events confirming the incorporation of the kanamycin selection marker gene. [75].

## 5. Haploid and Double Haploid Production of Jatropha

The use of gametes containing haploid chromosome number (n) for the development of the entire plantlet results in the production of haploid plants. Haploid production is essential for the generation of hybrids with high yield and oil along with disease resistance. Haploid production aids in the development of genetically homozygous plants from heterozygous parent plants and, in return, can serve as parents in crossbreeding. Gametic embryogenesis can be done to produce homozygous lines within a short duration when compared to conventional breeding methods that involve the selfing of several generations. Culturing of male gametophyte (androgenesis) and female gametophyte (gynogenesis) results in haploid embryo development. Double haploids can be generated by genome duplication either through spontaneous duplication or by using microtubule depolymerizing agents, such as colchicine and trifluralin [76].

The first successful anther culture of jatropha was reported by Madan et al., (2019). They induced callus from anthers of immature buds of jatropha. The anthers cultured on MS medium supplemented with 1.0 mg/L BA and picloram induced 77% callus. The obtained callus showed regeneration of plants on medium supplemented with 2.0 mg/L BA, 0.5 mg/L KN, and 0.5 mg/L NAA. Approximately 90% of the elongated shoots showed rooting on half-strength MS medium with 2.0 mg/L IBA. All the in-vitro plants derived from anther showed 100% success in primary hardening (grown under greenhouse conditions) and 85% in secondary hardening (field conditions). The embryogenic callus analyzed using molecular and flow cytometry showed that 5.7% of plantlets were haploid and 3% of the plantlets were double haploids [76].

For the development of haploid and double haploid plants, microspore culture is most preferred, especially in jatropha, as it contains more male flowers than female flowers. Shrivastava et al., (2021) reported microspore gametic embryogenesis for the first time in jatropha. They observed that when tetrads, early, mid-un-vacuolated, and vacuolated late-stage uninucleate microspores inoculated on modified MS medium supplemented with 2.0 mg/L 2,4-D, 0.1 mg/L KN, 300 mg/L casein hydrolysate, 1.0 g/L glutamine, 0.5 mg/L folic acid, 0.05 mg/L biotin, and 5% sucrose resulted in induction and formation of embryo-like structures (ELS). The cultures were incubated at 4 °C for seven days followed by incubation at 25 °C for 15 days and then under 15 °C for 10 days. The different developmental stages of microspore embryogenesis were confirmed by microscopic analysis. The established calli and ELSs were verified to be haploid by flow cytometric analysis [77]. Although the number of female flowers in jatropha is low, Lopez-Puc et al., (2021) developed homozygous lines of *J. curcas* by gynogenesis. They established a protocol for the development of in-vitro plants from unfertilized ovules of *J. curcas*. They reported that green friable gynogenic calli developed on MS medium supplemented with 6.66 µM BAP and 4.9 µM IBA when transferred to MS medium with 22.09 µM BAP and 3.40 µM paclobutrazol (PBZ) resulted in the formation of gynogenic embryo. These generated embryos were cultured on MS medium containing 2.22 µM BAP and 0.28 µM IAA for the shoot development. Root development occurred on half-strength MS medium supplemented with 18.65 µM IBA [78].

## 6. Clonal Fidelity Analysis of Jatropha Using Molecular Markers

Molecular markers help in accessing the genetic variation of in-vitro raised plants from that of parental plant. They also help in evaluating the biodiversity and phylogenetic relationships, generating genetic linkage maps, tagging, and mapping of useful traits of a plant specimen. Some of the widely studied molecular markers include RAPD (Random amplified polymorphic DNA), AFLP (amplified fragment length polymorphism), ISSRs (inter simple sequence repeats), SSRs (simple sequence repeats), and SNPs (single-nucleotide polymorphism), etc. [79].

In-vitro regeneration and molecular characterization studies were performed on *J. curcas* by El-Sayed et al., (2020). Molecular characterization to determine genetic variation between regenerated, micro propagated, and mother plants was performed using RAPD and ISSR analyses. Their RAPD results revealed that out of 117 amplified products, 25 were polymorphic, indicating 21.3% polymorphism. In contrast, their ISSR results showed 22 polymorphic bands out of 116 scorable bands, indicating 18.96% polymorphism [80]. *J. curcas* tissue culture regenerates (TCR) obtained using nodal/apical shoot segments and leaves as explants were evaluated at different passages of subculture for their genetic homogeneity using RAPD, ISSR, SSR, and flow cytometry by Rathore et al., (2014). They observed that both node and leaf explant derived TCR showed genetic homogeneity in the fifth generation using RAPD and ploidy level stability at the 20th generation using flow cytometry analysis. The TCR obtained using leaf explant showed genetic stability and ploidy level stability at 10th generation using ISSR markers and flow cytometry analysis, respectively. TCR of node and leaf explants showed genetic homogeneity using SSR markers at the 20th generation. This study supports the fact that the regeneration of organized meristem is genetically stable [81].

Molecular studies using RAPD and AFLP for differentiating toxic and non-toxic varieties of *J. curcas* were done by Sudheer Pamidimarri et al., (2009). They analyzed 371 RAPD and 1442 AFLP markers and found that 15.09% RAPD and 16.49% AFLP markers were specific to either of the varieties. They observed the genetic similarity between toxic and non-toxic varieties to be 0.92 by RAPD and 0.90 by AFLP analysis, suggesting that both techniques were equally competitive in detecting polymorphic markers [82]. The use of different molecular markers allows a better opportunity for the identification of genetic variations in *J. curcas.* These marker studies also aid in the selection and development of high yield cultivars for use in plant breeding programs.

## 7. Conclusions and Future Prospects

In this fast-moving world, humans are growing, evolving, and seeking to construct a sustainable and eco-friendly planet for future generations. At the same time, the world is looking forward to achieve the pinnacle of industrialization and automotive designing for improved quality of living. To make these things work together, we must be prepared to use environmentally friendly fuels in order to create a pollution-free atmosphere conducive to a healthy lifestyle. Biodiesel and similar biofuels derived from plants such as jatropha will be an excellent substitute for pollution-free emissions.

In conclusion, we have discussed various tissue culture techniques employed for the regeneration of the elite germplasm through both direct and indirect organogenesis and somatic embryogenesis, development of haploids using androgenesis and gynogenesis, and subsequent di-haploids for enhanced biomass yield that will help in meeting the demand of the biomass. Different gene modifications have been made for obtaining the increased oil content and other agronomic characteristics of interest. There are several scientific studies that back up the notion of employing biotechnology tools to regenerate and multiply jatropha. As a result, the scientific community should optimize large-scale production protocols and validate them for the future, as well as adapt molecular marker techniques, such as RAPD and ISSR, for maintaining the genetic stability of elite germplasms for a long-term supply of jatropha biomass. Adapting modern tools like CRISPR technology and editing the desired genes for optimal biodiesel production in jatropha will help to meet the demand for biodiesel production.

## Figures and Tables

**Table 2:** Indirect organogenesis from various explants of *Jatropha curcas* L.

Explant	Callusing Media	Callusing Response	Shooting Media	Shooting Response/No. of Shoots	Rooting Media	Rooting Response/No. of Roots	Percentage of Acclimatization	Reference
Leaf	MS + 2.22 µM BAP + 4.9 µM IBA	100%	MS + 2.22 µM BAP + 2.46 µM IBA	67% /10.7 shoots	Full strength MS medium	88%	More than 80%	[14]
Leaf	MS + 1.0 mg/L NAA + 5.0 mg/L BAP (compact green)	-	MS + 1.5 mg/L BAP + 0.5 mg/L IBA	12.62 ± 0.24 buds	MS + 3.0 mg/L IBA	-	-	[42]
Leaf	MS + 3–5 µM IBA + 27.0 µM BAP	-	MS + 27.0 µM BAP + 3.0 µM IBA	10 ± 2.35 buds	Basal MS medium	-	-	[46]
Leaf	MS + 1.0 mg/L BAP + 0.50 mg/L NAA	80%	MS + 2.0 mg/L BAP + 0.5 mg/L NAA	60%/4 shoots	Basal MS medium	90%	70%	[47]
Leaf	MS + 0.25 mg/L BAP + 0.25 mg/L NAA or MS + 0.50 mg/L BAP + 0.50 mg/L NAA	90%	MS + 2.5 mg/L BAP + 0.5 mg/L NAA	70%/5 shoots	Basal MS medium	95%	70%	[43]
Leaf	MS + 2.0 mg/L BAP + 1.0 mg/L IBA	90.5%	MS + 2.0 mg/L BAP	43.7%/20.00 ± 1.23 shoots	1/2 MS + 0.5 mg/L IBA + 100 mg/L phloroglucinol	-	-	[44]
Petiole	MS + 4.44 µM BAP + 2.45 µM IBA	95.9 ± 1.79%	MS + 2.22 µM BAP + 8.56 µM IAA	10–13 shoots	MS + 2.45 µM IBA + 0.54 µM NAA	More than 72%	More than 98%	[48]
Petiole segments	MS + 2.22 µM BAP + 4.90 µM IBA	-	MS + 4.44 µM BAP + 2.46 µM IBA	5.4 ± 1.4 shoots	Basal MS medium	85.71%	82%	[37]
Cotyledonary leaves	MS + 0.45 µM TDZ	100%	MS + 0.45 µM TDZ. Proliferation medium: MS + 8.88 µM BAP + 54.3 µM AdS.	82.67%/11.9 shoots	½ MS + 1.47 µM IBA	61.66%/8.6 roots	-	[45]
Cotyledon	MS + 1.5 mg/L BAP + 0.10 mg/L IBA	-	MS + 2.0 mg/L BAP + 0.05 mg/L IBA + 0.5 GA_3_	45.78%	-	-	-	[49]
Embryo	MS + 1.5 mg/L BAP + 1.0 mg/L IBA	-	Same as callusing medium.	5 to 6 shoots	½ MS + 3.0 mg/L IBA	4–5 roots	95%	[39]
Node	MS + 8.0 µM BAP + 2.0 µM IBA	-	Same as callusing medium.	7.2 ± 0.84	½ MS + 2.0 µM IBA	5.6 ± 0.55 roots	80%	[40]

**Table 3:** Somatic embryogenesis from various explants of *Jatropha curcas* L.

Explant	Media and Plant Growth Regulators	Response	Percentage of Embryogenesis	No. of Embryos	Percentage of Acclimatization	Reference
Leaf	MS + 2.3 µM KN + 1.0 µM IBA + 13.6 µM AdS	Globular somatic embryo development	80%	58.5 ± 12.7	90%	[53]
Cotyledonary leaves	Initiation medium: MS + 0.2 mg/L IAA. Subculture medium: MS + 0.2 mg/L IAA + 1.5 mg/L BAP	Embryo callus transformed to heart, torpedo shaped embryos	84.6 ± 1.5%	-	50%	[58]
Leaf and shoot tip	Embryogenesis induction medium: MS + 0.5 mg/L 2,4-D + 5.0 mg/L BAP + 684.2 µM glutamine + 520.5 µM citric acid. Embryo to plant conversion medium: MS + 2.0 mg/L BAP + 684.2 µM glutamine + 520.5 µM citric acid.	Green embryogenic callus	-	Plant conversion rate is 51 ± 0.9 for leaf and 54 ± 0.6 for shoot tip explant	25 to 30%	[52]
Cotyledon and embryo axis	MS + 1.0 mg/L picloram	Somatic embryos development	65% and 45%	-	-	[57]
Immature zygotic embryos	Direct somatic embryogenesis: MS + 0.1–0.2 mg/L 2,4-D. Indirect somatic embryogenesis: B5 vitamins rich medium.	-	57.3 ± 8.7%	-	95%	[54]
Foliar leaves	Embryogenesis Induction medium: MS + 5.0 µM BAP. Embryo maturation medium: MS + 8.87 µM BA	Direct somatic embryogenesis from leaf margins	-	-	-	[59]
Petiole	Embryogenic callus induction medium: Y3 medium + 0.5 mg/L 2,4-D + 0.5 mg/L BAP Somatic embryos induction medium: Y3 medium + 0.05 mg/L 2,4-D +2.0 mg/L BAP. Embryos maturation: ½ MS + 2.0 mg/L BAP	Somatic embryos were differentiated and matured in auxin free medium	100%	-	-	[55]
Cotyledon	MS + 2.0 mg/L BAP	Globular somatic embryo development	90%	40 embryos per explant	-	[56]

**Table 4:** Various acclimatization media for establishment of regenerated plantlets.

Acclimatization Media Composition	Reference
Soil rite	[38]
Soil and vermiculite (1:1)	[62]
Garden soil, vermiculite, sand (1:1:1)	[28]
Sand and soil (1:1)	[17,18,22,31]
Garden soil, sand, manure (1:1:1)	[41]
Garden soil, cocopeat, sand, vermicompost (1:1:1:1)	[63]

## Data Availability

Not applicable.
